# Association between long non-coding RNA polymorphisms and cancer risk: a meta-analysis

**DOI:** 10.1042/BSR20180365

**Published:** 2018-07-31

**Authors:** Xin Huang, Weiyue Zhang, Zengwu Shao

**Affiliations:** 1 Department of Orthopaedics, Union Hospital, Tongji Medical College, Huazhong University of Science and Technology, Wuhan 430022, China; 2 Department of Endocrinology, Union Hospital, Tongji Medical College, Huazhong University of Science and Technology, Wuhan 430022, China

**Keywords:** Cancer, LncRNA, Polymorphisms

## Abstract

Several studies have suggested that long non-coding RNA (lncRNA) gene polymorphisms are associated with cancer risk. In the present study, we conducted a meta-analysis related to studies on the association between lncRNA single-nucleotide polymorphisms (SNPs) and the overall risk of cancer. A total of 12 SNPs in five common lncRNA genes were finally included in the meta-analysis. In the lncRNA antisense non-coding RNA (ncRNA) in the INK4 locus (ANRIL), the rs1333048 A/C, rs4977574 A/G, and rs10757278 A/G polymorphisms, but not rs1333045 C/T, were correlated with overall cancer risk. Our study also demonstrated that other SNPs were correlated with overall cancer risk, namely, metastasis-associated lung adenocarcinoma transcript 1 (MALAT1, rs619586 A/G), HOXA distal transcript antisense RNA (HOTTIP, rs1859168 A/C), and highly up-regulated in liver cancer (HULC, rs7763881 A/C). Moreover, four prostate cancer-associated ncRNA 1 (PRNCR1, rs16901946 G/A, rs13252298 G/A, rs1016343 T/C, and rs1456315 G/A) SNPs were in association with cancer risk. No association was found between the PRNCR1 (rs7007694 C/T) SNP and the risk of cancer. In conclusion, our results suggest that several studied lncRNA SNPs are associated with overall cancer risk. Therefore, they might be potential predictive biomarkers for the risk of cancer. More studies based on larger sample sizes and more lncRNA SNPs are warranted to confirm these findings.

## Introduction

As a new class of functional non-coding RNAs (ncRNAs), long ncRNAs (lncRNAs) are made up of over 200 nts and lack the ability of protein coding [1]. Recently, the association between lncRNA and human diseases, especially cancer, has been widely investigated. Compared with other ncRNAs, lncRNAs play an important role in numerous vital activities of cell, including the regulation of epigenetic modifications, cell cycle, cell differentiation, and stress response [2]. The most important function of lncRNA is involvement in the tumorigenesis as proto-oncogene [3] or anti-oncogene [4]. Moreover, the differential expression of lncRNA may facilitate tumor cell proliferation, invasion, and metastasis [5].

Currently, single nucleotide polymorphisms (SNPs) are the most common genetic variants of concern and universally present in lncRNA genes. It is predicted that the expression and function of lncRNAs are affected by SNPs [6]. Studies have also suggested that polymorphism in lncRNA may influence the process of splicing and stability of mRNA conformation, leading to the modification of their interacting partners [7]. To date, several studies have assessed the associations amongst more than 20 lncRNA polymorphisms and susceptibility of cancers, but the results are inconsistent.

In the present study, we conducted a meta-analysis of epidemiological studies to explore the associations between five lncRNA SNPs and overall cancer risk. Furthermore, our study may shed some light on the biomarkers for predicting cancer risk.

## Materials and methods

### Publication search

A computerized literature search was performed in the Medline, PubMed, Web of Science, and Embase database up to 6 Februrary 2018. The search strategy included the terms (‘lncRNA’ or ‘long non-coding RNA’) and (‘polymorphisms’ or ‘variants’ or ‘variation’ or ‘SNP’) and (‘cancer’ or ‘carcinoma’ or ‘tumor’ or ‘neoplasm’). To be eligible for inclusion in the meta-analysis, a study must meet the following criteria: (i) case–control study or cohort study; (ii) assessing the association between lncRNA SNPs and cancer risk; (iii) having an available genotype or allele frequency for estimating an odds ratio (OR) with 95% confidence interval (95% CI) or hazard ratio (HR) with 95% CI; and (iv) genotype frequencies in controls being consistent with those expected from Hardy–Weinberg equilibrium (HWE) (*P*>0.05). The exclusion criteria were: (i) duplicate studies; (ii) not relevant to cancer or lncRNA SNPs; or (iii) no available data and the authors could not be contacted.

### Data extraction and quality assessment

Two investigators (X.H. and W.Z.) evaluated the eligibility of all retrieved studies and extracted the relevant data independently. Extracted databases were then cross-checked between the two authors to rule out any discrepancy. Disagreement was resolved by consulting with the third investigator (Z.S.). The study quality was assessed in accordance with the Newcastle–Ottawa Scale (NOS) (Supplementary Table S1). Eight items were extracted, and each item scored 1. The total scores ranged from 0 to 8. If the scores were ≥7, then the study was considered to be of high quality.

### Statistical analysis

The statistical analysis was performed using STATA 14. Estimates were summarized as ORs with 95% CIs for each study (*P*<0.05 was considered statistically significant). The genotype frequencies of the lncRNA polymorphisms for the HWE were calculated for the controls using the chi-square test, and *P*<0.05 was considered as significant disequilibrium. The between-study heterogeneity was evaluated by using the chi-square test and the *I^2^* statistic. An *I^2^* value of >50% of the *I^2^* statistic was considered to indicate significant heterogeneity [8]. When a significant heterogeneity existed across the included studies, a random-effects model was used for the analysis. Otherwise, the fixed-effects model was used. Subgroup analyses were performed to detect the source of heterogeneity. As to genotype comparison, the risks of the heterozygote and variant homozygote compared with the wild-type homozygote were estimated respectively. Then we evaluated the dominant and recessive effects of the variant allele (heterozygote + variant homozygote compared with wild-type homozygote and variant homozygote compared with heterozygote + wild-type homozygote), respectively. Begg’s rank correlation and Egger’s linear regression method were used to assess the publication bias statistically. A two-tailed *P*-value <0.05 implies a statistically significant publication bias [9,10]. We further conducted sensitivity analyses to substantiate the stability of results and detect the potential source of heterogeneity.

## Results

### Characteristics of the eligible studies

**Figure 1:**
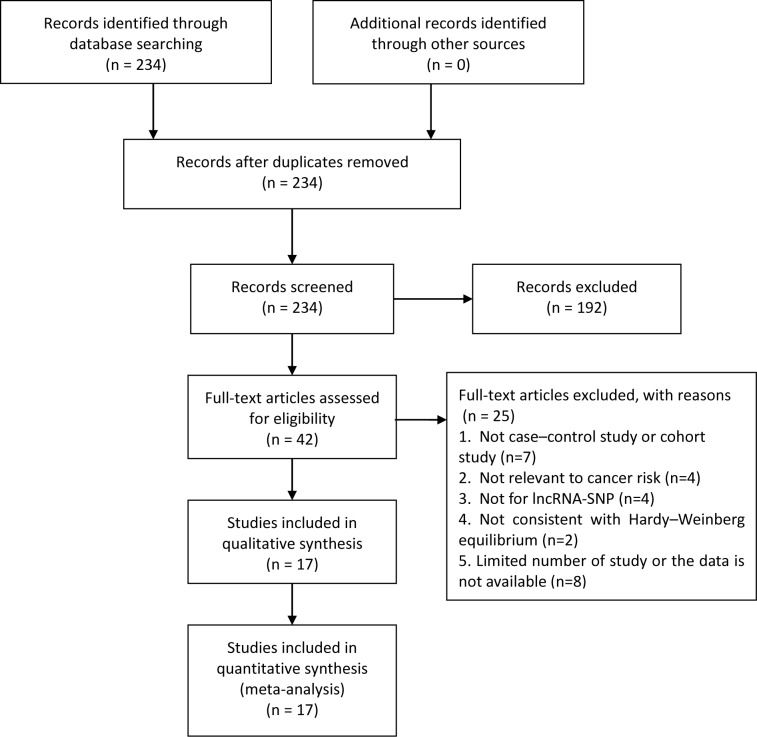
The studies identified in this meta-analysis based on the inclusion and exclusion criteria

Finally, a total of 234 articles were included in the meta-analysis, 42 case–control studies that met our inclusion criteria were included in quantitative synthesis, and 17 of them involving 9548 cases and 9828 controls were included in our meta-analysis (Figure 1). Table 1 lists the characteristics of the eligible studies. Amongst the 17 case–control studies, the control groups of 9 were hospital-based and 8 were population-based. Genotyping methods included tetra-primer amplification refractory mutation system (T-ARMS)-PCR (2), MALDI-TOF MS (1), PCR-restriction fragment length polymorphism (RFLP) (5), created restriction site (CRS)-RFLP (1), TaqMan (3), MassARRAY (4), multiplex PCR-based Invader assay (1), and SNPlex Genotyping System (1) (Table 1). Table 2 presents the genotype frequency distributions of a total 19 SNPs in five lncRNA genes (antisense ncRNA in the INK4 locus (*ANRIL*), metastasis-associated lung adenocarcinoma transcript 1 (*MALAT1*), HOXA distal transcript antisense RNA (*HOTTIP*), highly up-regulated in liver cancer (*HULC*), and prostate cancer-associated ncRNA 1 (*PRNCR1*)) involved in the 17 eligible studies. After removal of those records for which *P*_HWE_<0.05, seven SNPs were found to be only based on one single eligible study. They were ANRIL rs2151280, MALAT1 rs3200401, MALAT1 rs7927113, MALAT1 rs1194338, HOTTIP rs5883064, PRNCR1 rs7841060, and PRNCR1 rs7463708. Therefore, the remaining 12 lncRNA SNPs were included in our final calculation (Table 2).

**Table 1:** Characteristics of eligible studies

Number	First author	Year	Country	Ethnicity	Sample size		Source of control groups	Genotyping method	Adjusted factors	Citation
					Case	Control				
1	Khorshidi et al.	2017	Iran	Asian	122	200	PB	T-ARMS-PCR	Age	[11]
2	Kang et al.	2015	China	Asian	380	380	HB	MALDI-TOF MS	Age, sex, and drinking status	[12]
3	Taheri et al.	2017	Iran	Asian	125	220	PB	T-ARMS-PCR	Age, BMI, and smoking history	[13]
4	Peng et al.	2017	China	Asian	487	489	PB	PCR-RFLP, CRS-RFLP	Age	[14]
5	Liu et al.	2012	China	Asian	1300	1344	PB	TaqMan Assay-PCR	Age, sex, smoking rate, and HBV chronic infection	[15]
6	Li et al.	2017	China	Asian	821	857	HB	MassARRAY	Age, sex, BMI, smoking, and alcohol drinking	[16]
7	Gong et al.	2016	China	Asian	498	213	HB	MassARRAY	Age and sex	[17]
8	Hu et al.	2017	China	Asian	921	921	PB	TaqMan Assay-PCR	Age, sex, and area of residence	[18]
9	Shaker et al.	2017	Egypt	Caucasian	120	96	PB	TaqMan Assay-PCR	Age and sex	[19]
10	He et al.	2017	China	Asian	494	494	HB	MassARRAY	*Helicobacter pylori* infection rate, age, sex, and smoking and drinking status	[20]
11	Duan et al.	2017	China	Asian	470	470	HB	PCR-RFLP	Age, sex, and drinking	[6]
12	Li et al.	2016	China	Asian	219	394	HB	PCR-RFLP	Age and sex	[21]
13	Sattarifard et al.	2017	Iran	Asian	178	180	HB	PCR-RFLP	Age	[22]
14	Li et al.	2013	China	Asian	313	595	HB	PCR-RFLP	Age and sex	[23]
15	Chung et al.	2010	Japan	Asian	1504	1554	HB	Multiplex PCR-based Invader	NM	[24]
16	Salinas et al.	2008	U.S.A.	Caucasian	1308	1266	PB	SNPlex genotyping system	Age	[25]
17	Zheng et al.	2010	China	Asian	288	155	PB	MassARRAY	Age, sex, and BMI	[26]

Abbreviations: BMI, body mass index; HB, hospital based; NM, not mentioned; PB, population based.

**Table 2:** Genotype frequency distributions of lncRNA SNPs studied in included studies

First author	Year	lncRNA	SNPs	Type of cancer	Sample size		Case			Control			*P* for HWE	Quality score
					Case	Control	Homozygote wild	Heterozygote	Homozygote variant	Homozygote wild	Heterozygote	Homozygote variant		
Khorshidi et al.	2017	ANRIL	rs1333045 (C/T)	Breast cancer	122	200	31	52	39	57	100	43	0.944	7
		ANRIL	rs1333048 (A/C)	Breast cancer	122	200	39	51	32	51	97	52	0.672	
		ANRIL	rs4977574 (A/G)	Breast cancer	122	200	61	44	17	81	93	26	0.931	
		ANRIL	rs10757278 (A/G)	Breast cancer	122	200	38	62	22	74	100	26	0.387	
Kang et al.	2015	ANRIL	rs2151280 (C/T)^1^	ESCC	380	380	57	153	161	43	173	154	0.595	8
Taheri et al.	2017	ANRIL	rs1333045 (C/T)	Prostate cancer	125	220	41	61	23	75	102	43	0.435	7
		ANRIL	rs1333048 (A/C)	Prostate cancer	148	220	25	65	58	101	88	31	0.103	
		ANRIL	rs4977574 (A/G)	Prostate cancer	114	220	55	46	13	79	109	32	0.570	
		ANRIL	rs10757278 (A/G)	Prostate cancer	132	220	14	65	53	95	93	32	0.241	
Peng et al.	2017	MALAT1	rs3200401 (T/C)^1^	Breast cancer	487	489	357	120	10	338	145	6	0.057	8
		MALAT1	rs619586 (A/G)	Breast cancer	487	489	415	65	7	386	93	10	0.124	
		MALAT1	rs7927113 (A/G)^1^	Breast cancer	487	489	476	10	1	469	19	1	0.096	
Liu et al.	2012	MALAT1	rs619586 (A/G)	HCC	1300	1344	1094	169	5	1115	205	10	0.864	8
Li et al.	2017	MALAT1	rs1194338 (A/C)^1^	CRC	821	857	389	357	72	381	377	95	0.905	8
Gong et al.	2016	HOTTIP	rs5883064 (C/T)^1^	Lung cancer	491	206	161	252	78	89	87	30	0.252	8
		HOTTIP	rs1859168 (A/C)	Lung cancer	491	210	151	254	86	85	94	31	0.549	
Hu et al.	2017	HOTTIP	rs1859168 (A/C)	Pancreatic cancer	921	921	239	497	185	364	421	136	0.428	8
Duan et al.	2017	HOTTIP	rs1859168 (A/C)	Gastric cancer	455	451	141	117	191	102	210	139	0.185	8
Kang et al.	2015	HULC	rs7763881 (A/C)	ESCC	380	380	122	168	84	99	195	81	0.412	8
Shaker et al.	2017	HULC	rs7763881 (A/C)	CRC	120	96	32	88	0	12	84	0	0.000^2^	6
Liu et al.	2012	HULC	rs7763881 (A/C)	HCC	1300	1344	377	617	283	333	695	288	0.057	8
He et al.	2017	PRNCR1	rs16901946 (A/G)	Gastric cancer	494	494	261	203	30	301	176	17	0.153	8
		PRNCR1	rs13252298 (A/G)	Gastric cancer	494	494	236	215	43	209	235	50	0.173	
		PRNCR1	rs7463708 (G/T)^1^	Gastric cancer	494	494	241	209	44	228	209	57	0.390	
		PRNCR1	rs7007694 (C/T)	Gastric cancer	494	494	264	199	31	272	198	24	0.111	
Li et al.	2016	PRNCR1	rs16901946 (A/G)	Gastric cancer	219	394	125	92	2	230	135	29	0.144	8
		PRNCR1	rs13252298 (A/G)	Gastric cancer	219	394	88	107	24	198	161	35	0.781	
		PRNCR1	rs7007694 (C/T)	Gastric cancer	219	394	142	72	5	214	159	21	0.219	
		PRNCR1	rs1016343 (C/T)	Gastric cancer	219	394	78	109	32	140	176	78	0.096	
		PRNCR1	rs1456315 (A/G)	Gastric cancer	219	394	109	103	7	179	177	38	0.546	
Sattarifard et al.	2017	PRNCR1	rs13252298 (A/G)	Prostate cancer	178	179	33	107	38	25	141	13	0.000^2^	7
		PRNCR1	rs1456315 (A/G)	Prostate cancer	178	180	30	148	0	92	88	0	0.000^2^	
		PRNCR1	rs7007694 (C/T)	Prostate cancer	178	180	150	28	0	139	41	0	0.085	
		PRNCR1	rs7841060 (G/T)^1^	Prostate cancer	178	180	29	149	0	96	84	0	0.000^2^	
Li et al.	2013	PRNCR1	rs1016343 (C/T)	CRC	313	595	117	156	40	227	276	92	0.593	8
		PRNCR1	rs13252298 (A/G)	CRC	313	595	166	121	26	264	270	61	0.508	
		PRNCR1	rs16901946 (A/G)	CRC	313	595	175	117	138	338	232	257	0.000^2^	
		PRNCR1	rs1456315 (A/G)	CRC	313	595	167	119	27	294	262	39	0.055	
		PRNCR1	rs7007694 (C/T)	CRC	313	595	184	107	22	362	208	25	0.474	
Chung et al.	2010	PRNCR1	rs1016343 (C/T)	Prostate cancer	1504	1554	650	667	185	841	608	103	0.624	7
		PRNCR1	rs13252298 (A/G)	Prostate cancer	1504	1554	808	556	137	609	737	204	0.416	
		PRNCR1	rs16901946 (A/G)	Prostate cancer	1504	1554	690	637	177	783	645	126	0.671	
		PRNCR1	rs1456315 (A/G)	Prostate cancer	1504	1554	905	495	104	663	703	187	0.975	
		PRNCR1	rs7007694 (C/T)	Prostate cancer	1504	1554	656	650	191	700	684	170	0.880	
Salinas et al.	2008	PRNCR1	rs1456315 (A/G)	Prostate cancer	1308	1266	464	598	192	401	605	227	0.964	7
		PRNCR1	rs1016343 (C/T)	Prostate cancer	1253	1233	711	454	88	796	385	52	0.529	
Zheng et al.	2010	PRNCR1	rs1016343 (C/T)	Prostate cancer	284	147	76	159	49	66	65	16	0.999	7

Abbreviations: CRC, colorectal cancer; EOC, epithelial ovarian cancer; ESCC, esophageal squamous cell carcinoma; HCC, hepatocellular carcinoma.

1 Not included due to the limited number of studies for this lncRNA locus.

2 Not included because the *P* of the HWE was <0.05.

### Quantitative data synthesis of 12 SNPs in five highly studied lncRNA genes

#### Four SNPs in ANRIL

First, we calculated the pooled ORs of all eligible studies to estimate the association between the four SNPs in ANRIL and overall cancer risk. The rs1333045 C/T polymorphism was not associated with cancer; and the rs1333048 A/C, rs4977574 A/G, and rs10757278 A/G polymorphisms were associated with overall cancer risk. The rs1333048 A/C polymorphism was associated with increased overall risk of cancer in all genetic models (C compared with A: *P*=0.000, OR = 2.06, 95% CI = 1.64–2.57; CC compared with AA: *P*=0.000, OR = 4.26, 95% CI = 2.67–6.78; AC compared with AA: *P*=0.049, OR = 1.45, 95% CI = 1.00–2.10; dominant model: *P*=0.001, OR = 1.80, 95% CI = 1.28–2.51; recessive model: *P*=0.000, OR = 2.01, 95% CI = 1.42–2.84). For the rs4977574 A/G polymorphism, both the heterozygote type AG and the dominant model were associated with decreased overall risk of cancer compared with the wild-type AA (AG compared with AA: *P*=0.006, OR = 0.62, 95% CI = 0.44–0.87; dominant model: *P*=0.007, OR = 0.64, 95% CI = 0.46–0.88). However, both the mutation type GG and the allelic model were associated with increased overall risk of cancer (GG compared with AA: *P*=0.000, OR = 2.40, 95% CI = 1.60–3.59; G compared with A: *P*=0.000, OR = 1.68, 95% CI = 1.35–2.08). For the rs10757278 A/G polymorphism, the heterozygote type AG, the dominant model, and the recessive model were associated with increased overall risk of cancer (AG compared with AA: *P*=0.000, OR = 2.13, 95% CI = 1.45–3.12; dominant model: *P*=0.000, OR = 2.58, 95% CI = 1.80–3.69; recessive model: *P*=0.000, OR = 2.64, 95% CI = 1.79–3.88). Nevertheless, the allelic model was associated with decreased overall risk of cancer (G compared with A: *P*=0.030, OR = 0.77, 95% CI = 0.60–0.97, Table 3).

**Table 3:** Meta-analysis of the association between common SNPs and cancer risk

Stratification	*n*	Allelic model			Mutation homozygote compared with wild-type			Heterozygote compared with wild-type			Dominant model			Recessive model		
		OR (95% CI)	*P*	*I^2^* (%)	OR (95% CI)	*P*	*I^2^* (%)	OR (95% CI)	*P*	*I^2^* (%)	OR (95% CI)	*P*	*I^2^* (%)	OR (95% CI)	P	I^2^ (%)
**ANRIL**																
rs1333048 (A/C)	2	**2.06 (1.64– 2.57)**	**0.000****^1^**	**94.3**	**4.26 (2.67– 6.78)**	**0.000****^1^**	**93.1**	**1.45 (1.00– 2.10)**	**0.049****^1^**	**93.0**	**1.80 (1.28– 2.51)**	**0.001**^1^	**95.7**	**2.01 (1.42– 2.84)**	**0.000**^a^	**92.7**
rs4977574 (A/G)	2	**1.68 (1.35– 2.08)**	**0.000**^1^	**96.7**	**2.40 (1.60– 3.59)**	**0.000**^1^	**96.1**	**0.62 (0.44– 0.87)**	**0.006**	**0.0**	**0.64 (0.46– 0.88)**	**0.007**	**0.0**	0.91 (0.57– 1.46)	0.693	0.0
rs10757278 (A/G)	2	**0.77 (0.60– 0.97)**	**0.030**	**0.0**	0.72 (0.43– 1.18)	0.192	0.0	**2.13 (1.45– 3.12)**	**0.000****^1^**	**90.7**	**2.58 (1.80– 3.69)**	**0.000**^1^	**93.9**	**2.64 (1.79– 3.88)**	**0.000****^1^**	**82.7**
rs1333045 (C/T)	2	1.15 (0.92– 1.43)	0.236	27.7	1.29 (0.83– 1.99)	0.260	28.5	1.03 (0.71– 1.48)	0.874	0.0	1.11 (0.79– 1.56)	0.556	0.0	1.30 (0.89– 1.88)	0.175^1^	60.4
**MALAT1**																
rs619586 (A/G)	2	**0.77 (0.65– 0.92)**	**0.003**	**9.7**	0.58 (0.28– 1.20)	0.141	0.0	**0.78 (0.65– 0.94)**	**0.009**	**33.5**	**0.77 (0.64– 0.92)**	**0.004**^1^	**27.9**	0.61 (0.30– 1.26)	0.180	0.0
**HOTTIP**																
rs1859168 (A/C)	3	**1.32 (1.19– 1.45)**	**0.000**^1^	**75.2**	**1.54 (1.27– 1.87)**	**0.000**^1^	**81.8**	**1.24 (1.06– 1.45)**	**0.006**^1^	**96.4**	**1.37 (1.19– 1.59)**	**0.000**^1^	**94.3**	**1.49 (1.26– 1.76)**	**0.000**	**0.0**
**HULC**																
rs7763881 (A/C)	3	**0.91 (0.83– 0.99)**	**0.040**	**0.0**	0.86 (0.71– 1.05)	0.132	0.0	**0.74 (0.63– 0.86)**	**0.000**	**41.3**	**0.77 (0.66– 0.89)**	**0.000**	**45.2**	1.02 (0.87– 1.21)	0.776	0.0
**PRNCR1**																
rs16901946 (G/A)	3	**1.15 (1.06– 1.25)**	**0.001**^1^	**66.4**	**1.26 (1.06–1.50)**	**0.008**^1^	**82.6**	**1.15 (1.03– 1.28)**	**0.017**	**0.0**	**1.17 (1.06– 1.30)**	**0.003**	**21.6**	**1.21 (1.03–1.43)**	**0.019**^1^	**81.7**
Type of cancer																
Gastric cancer	2	1.15 (0.97– 1.35)	0.104^1^	83.8	0.96 (0.59– 1.56)	0.871^1^	92.4	**1.30 (1.06– 1.60)**	**0.013**	**0.0**	**1.26 (1.03– 1.54)**	**0.025**	**40.7**	0.86 (0.53– 1.39)	0.533^1^	92.4
rs13252298 (G/A)	4	**0.78 (0.72– 0.85)**	**0.000**^1^	**89.2**	**0.68 (0.56– 0.81)**	**0.000**^1^	**81.6**	**0.69 (0.62– 0.77)**	**0.000**^1^	**85.1**	**0.81 (0.73– 0.90)**	**0.000**^1^	**73.7**	0.85 (0.72– 1.01)	0.065^1^	82.7
**Type of cancer**																
Gastric cancer	2	1.00 (0.86– 1.16)	0.994^1^	86.6	0.99 (0.69– 1.41)	0.945^1^	72.1	1.01 (0.82– 1.25)	0.923^1^	86.7	1.01 (0.83– 1.23)	0.945^1^	88.5	0.98 (0.70– 1.38)	0.921	21.1
rs7007694 (C/T)	5	1.03 (0.95– 1.12)	0.522^1^	69.0	1.19 (0.98– 1.45)	0.086^1^	58.4	0.96 (0.86– 1.07)	0.443	42.5	0.99 (0.89– 1.10)	0.848^1^	61.0	1.19 (0.98– 1.44)	0.070	49.9
**Type of cancer**																
Gastric cancer	2	0.92 (0.78– 1.09)	0.332^1^	85.8	0.92 (0.58– 1.47)	0.730^1^	80.4	0.89 (0.73– 1.10)	0.280^1^	71.6	0.89 (0.73– 1.09)	0.269^1^	81.7	0.96 (0.60– 1.52)	0.853^1^	75.0
Prostate cancer	2	1.05 (0.95– 1.16)	0.371^1^	69.6	1.20 (0.95– 1.51)	0.126		0.98 (0.85– 1.13)	0.769^1^	64.0	1.02 (0.88– 1.16)	0.832^1^	69.2	1.19 (0.96– 1.48)	0.120	
rs1016343 (T/C)	5	**1.31 (1.22– 1.41)**	**0.000**^1^	**85.2**	**1.67 (1.41– 1.97)**	**0.000**^1^	**86.0**	**1.35 (1.22– 1.49)**	**0.000**	**47.2**	**1.41 (1.28– 1.55)**	**0.000**^1^	**73.1**	**1.42 (1.21– 1.66)**	**0.000**^1^	**84.5**
**Ethnicity**																
Asian	4	**1.30 (1.19– 1.41)**	**0.000**^1^	**88.7**	**1.60 (1.33– 1.94)**	**0.000**^1^	**89.3**	**1.37 (1.21– 1.54)**	**0.000**^1^	**59.8**	**1.42 (1.26– 1.59)**	**0.000**^1^	**79.8**	**1.35 (1.13– 1.61)**	**0.001**^1^	**87.7**
Type of cancer																
Prostate cancer	3	**1.45 (1.34– 1.57)**	**0.000**	**1.9**	**2.21 (1.81– 2.70)**	**0.000**	**0.0**	**1.41 (1.27– 1.57)**	**0.000**	**49.3**	**1.51 (1.37– 1.68)**	**0.000**^1^	**55.6**	**1.86 (1.54– 2.26)**	**0.000**	**0.0**
rs1456315 (G/A)	4	**0.77 (0.72– 0.83)**	**0.000**^1^	**94.6**	**0.59 (0.49– 0.69)**	**0.000**^1^	**85.5**	**0.76 (0.68– 0.83)**	**0.000**^1^	**95.4**	**0.72 (0.66– 0.79)**	**0.000**^1^	**95.7**	**0.69 (0.59– 0.81)**	**0.000**^1^	**80.8**
**Ethnicity**																
Asian	3	**0.72 (0.66– 0.79)**	**0.000**^1^	**95.7**	**0.48 (0.39– 0.60)**	**0.000**^1^	**86.4**	**0.71 (0.63– 0.80)**	**0.000**^1^	**96.4**	**0.68 (0.61– 0.76)**	**0.000**^1^	**96.6**	**0.60 (0.49– 0.75)**	**0.000**^1^	**84.2**
Type of cancer																
Prostate cancer	2	**0.75 (0.70– 0.81)**	**0.000**^1^	**97.2**	**0.56 (0.47– 0.67)**	**0.000**^1^	**90.7**	**0.73 (0.66– 0.82)**	**0.000**^1^	**97.6**	**0.69 (0.63– 0.77)**	**0.000**^1^	**97.8**	**0.68 (0.58– 0.80)**	**0.000**^1^	**81.6**

The results are in bold if *P*<0.05.

1 *P* was calculated by random model.

#### One SNP in MALAT1

The meta-analysis showed that MALAT1 rs619586 A/G polymorphism was associated with overall cancer risk. For the rs619586 A/G polymorphism, the allelic model, the heterozygote type AG and the dominant model were associated with decreased overall risk of cancer compared with the wild-type AA (G compared with A: *P*=0.003, OR = 0.77, 95% CI = 0.65–0.92; AG compared with AA: *P*=0.009, OR = 0.78, 95% CI = 0.65–0.94; dominant model: *P*=0.004, OR = 0.77, 95% CI = 0.64–0.92, Table 3).

#### One SNP in HOTTIP

Our results suggested that the HOTTIP rs1859168 A/C polymorphism was associated with increased overall risk of cancer in all genetic models (C compared with A: *P*=0.000, OR = 1.32, 95% CI = 1.19–1.45; CC compared with AA: *P*=0.000, OR = 1.54, 95% CI = 1.27–1.87; AC compared with AA: *P*=0.006, OR = 1.24, 95% CI = 1.06–1.45; dominant model: *P*= 0.000, OR = 1.37, 95% CI = 1.19–1.59; recessive model: *P*=0.000, OR = 1.49, 95% CI = 1.26–1.76, Table 3).

#### One SNP in HULC

In the present study, the allelic model, the heterozygote type AC, and the dominant model of HULC rs7763881 A/C polymorphism were associated with decreased overall risk of cancer compared with the wild-type AA (C compared with A: *P*=0.040, OR = 0.91, 95% CI = 0.83–0.99; AC compared with AA: *P*=0.000, OR = 0.74, 95% CI = 0.63–0.86; dominant model: *P*=0.000, OR = 0.77, 95% CI = 0.66–0.89, Table 3).

#### Five SNPs in PRNCR1

The pooled OR and stratified analyses showed that amongst the five PRNCR1 SNPs included in the meta-analysis, only rs16901946 G/A, rs13252298 G/A, rs1016343 T/C, and rs1456315 G/A were associated with cancer risk, while the association of the rs7007694 C/T was not statistically significant (*P*>0.05).

The rs16901946 G/A polymorphism was associated with increased overall risk of cancer in all genetic models (A compared with G: *P*=0.001, OR = 1.15, 95% CI = 1.06–1.25; AA compared with GG: *P*=0.008, OR = 1.26, 95% CI = 1.06–1.50; AG compared with GG: *P*=0.017, OR = 1.15, 95% CI = 1.03–1.28; dominant model: *P*=0.003, OR = 1.17, 95% CI = 1.06–1.30; recessive model: *P*=0.019, OR = 1.21, 95% CI = 1.03–1.43).

For the rs13252298 G/A polymorphism, the allelic model, the mutation type AA, the heterozygote type AG, and the dominant model were associated with decreased overall risk of cancer compared with the wild-type GG (A compared with G: *P*=0.000, OR = 0.78, 95% CI = 0.72–0.85; AA compared with GG: *P*=0.000, OR = 0.68, 95% CI = 0.56–0.81; AG compared with GG: *P*=0.000, OR = 0.69, 95% CI = 0.62–0.77; dominant model: *P*=0.000, OR = 0.81, 95% CI = 0.73–0.90).

Additionally, the rs1016343 T/C polymorphism was associated with increased overall risk of cancer in all genetic models (C compared with T: *P*=0.000, OR = 1.31, 95% CI = 1.22–1.41; CC compared with TT: *P*=0.000, OR = 1.67, 95% CI = 1.41–1.97; CT compared with TT: *P*=0.000, OR = 1.35, 95% CI = 1.22–1.49; dominant model: *P*=0.000, OR = 1.41, 95% CI = 1.28–1.55; recessive model: *P*=0.000, OR = 1.42, 95% CI = 1.21–1.66).

The rs1456315 G/A polymorphism was associated with decreased overall risk of cancer in all genetic models (A compared with G: *P*=0.000, OR = 0.77, 95% CI = 0.72–0.83; AA compared with GG: *P*=0.000, OR = 0.59, 95% CI = 0.49–0.69; AG compared with GG: *P*=0.000, OR = 0.76, 95% CI = 0.68–0.83; dominant model: *P*=0.000, OR = 0.72, 95% CI = 0.66–0.79; recessive model: *P*=0.000, OR = 0.69, 95% CI = 0.59–0.81, Table 3).

Due to heterogeneity, we performed stratified analyses based on ethnicity and cancer type. Stratified analyses based on cancer type showed a significant association between the rs16901946 G/A polymorphism and increased risk of gastric cancer in the heterozygote type AG and the dominant model. In the Asian subgroup, the rs1016343 T/C polymorphism was associated with increased cancer risk in all genetic models. When stratified with cancer type, a significant association between the rs1456315 G/A polymorphism and decreased risk of prostate cancer was observed in our study (Table 3).

### Heterogeneity

There was interstudy heterogeneity (slight, moderate, or severe) in the overall comparison and the subgroup analyses (Table 3). We subsequently performed sensitivity analyses to explore the influence of an individual study on the pooled results by estimating the sensitivity before and after the removal of the study from the analysis. Some ORs and 95% CIs ranged from insignificantly to statistically significant after individual studies were removed (Supplementary Table S2).

### Publication bias

We used Begg’s test and Egger’s test to evaluate potential publication bias of the included studies. No statistically significant publication bias was indicated in any of the genetic models for all lncRNA SNPs (Table 4).

**Table 4:** The results of Begg’s and Egger’s test for the publication bias

Comparison type	Begg’s test		Egger’s test	
	Z-value	*P*-value	Z-value	*P*-value
**ANRIL rs1333048 (A/C)**				
Allelic model	0.00	1.000	NA	NA
Mutation homozygote compared with wild-type	0.00	1.000	NA	NA
Heterozygote compared with wild-type	0.00	1.000	NA	NA
Dominant model	0.00	1.000	NA	NA
Recessive model	0.00	1.000	NA	NA
**ANRIL rs4977574 (A/G)**				
Allelic model	0.00	1.000	NA	NA
Mutation homozygote compared with wild-type	0.00	1.000	NA	NA
Heterozygote compared with wild-type	0.00	1.000	NA	NA
Dominant model	0.00	1.000	NA	NA
Recessive model	0.00	1.000	NA	NA
**ANRIL rs10757278 (A/G)**				
Allelic model	0.00	1.000	NA	NA
Mutation homozygote compared with wild-type	0.00	1.000	NA	NA
Heterozygote compared with wild-type	0.00	1.000	NA	NA
Dominant model	0.00	1.000	NA	NA
Recessive model	0.00	1.000	NA	NA
**ANRIL rs1333045 (C/T)**				
Allelic model	0.00	1.000	NA	NA
Mutation homozygote compared with wild-type	0.00	1.000	NA	NA
Heterozygote compared with wild-type	0.00	1.000	NA	NA
Dominant model	0.00	1.000	NA	NA
Recessive model	0.00	1.000	NA	NA
**MALAT1 rs619586 (A/G)**				
Allelic model	0.00	1.000	NA	NA
Mutation homozygote compared with wild-type	0.00	1.000	NA	NA
Heterozygote compared with wild-type	0.00	1.000	NA	NA
Dominant model	0.00	1.000	NA	NA
Recessive model	0.00	1.000	NA	NA
**HOTTIP rs1859168 (A/C)**				
Allelic model	0.00	1.000	−0.86	0.548
Mutation homozygote compared with wild-type	0.00	1.000	−0.46	0.725
Heterozygote compared with wild-type	0.00	1.000	−1.02	0.494
Dominant model	0.00	1.000	−0.91	0.531
Recessive model	0.00	1.000	−0.75	0.590
**HULC rs7763881 (A/C)**				
Allelic model	1.04	0.296	−3.13	0.197
Mutation homozygote compared with wild-type	0.00	1.000	NA	NA
Heterozygote compared with wild-type	1.04	0.296	−9.06	0.070
Dominant model	1.04	0.296	−5.60	0.113
Recessive model	0.00	1.000	NA	NA
**PRNCR1 rs16901946 (G/A)**				
Allelic model	0.34	0.734	−0.71	0.553
Mutation homozygote compared with wild-type	0.34	0.734	−0.71	0.553
Heterozygote compared with wild-type	−0.34	1.000	0.38	0.742
Dominant model	−0.34	1.000	−0.27	0.810
Recessive model	0.34	0.734	−0.19	0.867
**PRNCR1 rs13252298 (G/A)**				
Allelic model	1.22	0.221	3.30	0.046
Mutation homozygote compared with wild-type	1.71	0.086	3.34	0.044
Heterozygote compared with wild-type	0.24	0.806	1.07	0.363
Dominant model	0.73	0.462	0.70	0.535
Recessive model	1.71	0.086	1.82	0.166
**PRNCR1 rs7007694 (C/T)**				
Allelic model	0.73	0.462	−1.42	0.251
Mutation homozygote compared with wild-type	−0.34	1.000	−0.10	0.933
Heterozygote compared with wild-type	1.71	0.086	−1.96	0.145
Dominant model	1.22	0.221	−1.70	0.188
Recessive model	−0.34	1.000	−0.04	0.974
**PRNCR1 rs1016343 (T/C)**				
Allelic model	0.24	0.806	−0.87	0.450
Mutation homozygote compared with wild-type	0.24	0.806	−0.83	0.467
Heterozygote compared with wild-type	−0.24	1.000	0.25	0.820
Dominant model	−0.24	1.000	−0.15	0.888
Recessive model	0.73	0.462	−1.29	0.288
**PRNCR1 rs1456315 (G/A)**				
Allelic model	1.22	0.221	1.74	0.181
Mutation homozygote compared with wild-type	−0.24	1.000	0.27	0.810
Heterozygote compared with wild-type	1.71	0.086	2.07	0.130
Dominant model	1.71	0.086	2.10	0.127
Recessive model	−0.24	1.000	0.20	0.862

Abbreviation: NA, not available.

## Discussion

It is known to all that over 20 lncRNA polymorphisms are associated with susceptibility of cancer. In recent studies, most of meta-analyses were conducted to focus on the association between lncRNA HOTAIR [27,28] or lncRNA ZNRD1-AS1 [28] or lncRNA POLR2E [29] or lncRNA H19 [28,30] polymorphisms and cancer risk. For example, the study of Lv et al. [28] included only four common lncRNA genes such as *H19, HOTAIR, ZNRD1-AS1*, and *PRNCR1*. However, more lncRNA polymorphisms with larger sample sizes are warranted. Therefore, a total of 12 SNPs in five common lncRNA genes were finally included in our study. In addition, our study was the first meta-analysis to show the significant association between the lncRNA ANRIL, MALAT1, HOTTIP, and HULC polymorphisms and cancer risk. Compared with the studies of Lv et al. [28] and Chu et al. [29], we decided to include more eligible studies related to lncRNA PRNCR1 genes according to the inclusion and exclusion criteria. Therefore, we included a larger size of cancer patients with more SNPs of lncRNA PRNCR1 into our study to confirm the results. More importantly, discussions about underlying mechanisms of each gene and the related polymorphisms were included in our study. It might help readers better understand the function of different lncRNA genes in cancer. Our study provides theoretical bases and research clues for future studies.

### The ANRIL SNPs

Chromosome region 9p21 is a hotspot for disease-associated polymorphisms and encodes three tumor suppressors, namely p16^INK4a^, p14^ARF^, and p15^INK4b^, and the lncRNA ANRIL [31]. ANRIL is 3.8-kb long and expressed on the reverse strand. It has been shown to bind to and recruit polycomb repression complex 2 (PRC2) to repress the expression of p15^INK4B^ [32]. Further study showed that SNPs can disrupt ANRIL splicing and result in a circular transcript that is resistant to RNase digestion [7]. The circularized transcripts affect the normal function of ANRIL and INK4/ARF expression. For example, rs1333048 has been shown to be associated with the level of highly sensitive C-reactive protein (hsCRP), which is a biomarker for systemic inflammation [33] and breast cancer susceptibility [34]. And previous results have revealed that rs4977574 is significantly associated with the risk of coronary artery disease [35]. Moreover, rs10757278 has been reported to increase the ANRIL variant EU741058 expression which contains exons 1–5 of the long transcript [36]. In addition, this SNP might modulate the ANRIL binding site for the transcription factor STAT1, which in turn regulates ANRIL expression [37]. In conclusion, three SNPs in ANRIL (rs1333048 A/C, rs4977574 A/G, and rs10757278 A/G) can be used to determine cancer risk.

### The MALAT1 SNPs

MALAT1 is located in chromosome 11q13, which is over 8000 nts long. It is enriched in nuclear speckles in interphase cells and concentrates in mitotic interchromatin granule clusters. And it is co-localized with pre-mRNA-splicing factor SF2/ASF and CC3 antigen in the nuclear speckles [38]. It is reported that lncRNA MALAT1 could regulate the expression through modulating transcription and the processing of post-transcriptional pre-mRNA in various genes [39]. Zhuo et al. [40] suggested that rs619586 SNP could bind with *miR-214* directly and suppress the expression of MALAT1. Several studies revealed that MALAT1 has an elevated expression and was associated with a higher risk and poorer survival in many kinds of cancers [41]. Our study showed that MALAT1 rs619586 A/G polymorphism was potential predictive biomarker of overall cancer risk.

### The HOTTIP SNPs

HOTTIP is an antisense non-coding transcript located at the 5′-end of the *HOXA* gene cluster. The previous study showed that rs1859168 might change the expression level of HOTTIP by affecting transcription factor binding sites [17]. Furthermore, RNAfold web server also revealed that rs1859168 could alter the centroid secondary structure and minimum free energy. It might also influence the folding of HOTTIP and its function [17]. Further studies are warranted to explore the specific mechanisms. Our results suggested that the HOTTIP rs1859168 A/C polymorphism was associated with increased overall risk of cancer. Although the detailed mechanisms underlying the association of SNP in HOTTIP with cancer susceptibility are unclear, these findings could provide a new insight into understanding the genetic factors of cancer susceptibility and carcinogenesis.

### The HULC SNPs

The lncRNA HULC is approximately 1.6 k nucleotide long and contains two exons but not translated [42]. Some studies have reported that HULC is highly up-regulated in hepatocellular carcinoma (HCC) and colorectal cancer (CRC) that metastasized to livers [42,43]. Rs7763881 SNP changing from A to C in *HULC* gene was located in the 6p24.3 region. Based on the Hapmap database, all the SNPs in HULC are in high linkage disequilibrium (LD). For example, rs7763881 was in complete LD with rs1328867 (r^2^ = 1), which is located in the promoter region of HULC. Additionally, the wild-type allele T of rs1328867 is predicted to bind with some transcription factors including C-Myc [15]. It has been identified that C-Myc is critical in the regulation of the growth, differentiation, and apoptosis of both normal and neoplastic liver cells [44]. In conclusion, HULC rs7763881 A/C polymorphism was associated with decreased overall risk of cancer.

### The PRNCR1 SNPs

The lncRNA PRNCR1, also referred to as PCAT8 and CARLo3, is transcribed from the ‘gene desert’ region of chromosome 8q24 (128.14–128.28 Mb) [24]. It has been stated that PRNCR1 is involved in the development of prostate cancer by activating androgen receptor (AR) [45]. Moreover, lncRNA PRNCR1 SNPs were observed to be risk of diverse cancers [21–23]. It might affect the predicted secondary structure of *PRNCR1* mRNA, altering the stability of PRNCR1 or the mRNA conformation, and giving rise to the modification of its interacting partners [24]. All the PRNCR1 polymorphisms in the exon region might result in the mechanism [28]. More specific mechanisms are warranted to be explored in further studies. Amongst the five PRNCR1 SNPs included in our study, rs16901946 G/A, rs13252298 G/A, rs1016343 T/C, and rs1456315 G/A could be predictive biomarkers of cancer risk.

## Limitations

Although this meta-analysis revealed the significant association between lncRNA polymorphisms and cancer risk, however, some limitations still should be acknowledged. First, the number of subjects in the included studies is relatively small, which might result in a lack of statistical power and prevent a meaningful analysis of the results. Second, in stratified analyses based on ethnicity and cancer type, we failed to perform further subgroup analysis because of limited relevant reports. Third, only English articles were included in our study and it may result in publication bias. Finally, study of the association between lncRNA polymorphisms and cancer risk remains an emerging field, we concluded only representative SNPs in our study. Therefore, additional prospective studies with larger sample sizes including other polymorphisms are warranted.

## Summary and future directions

We systematically reviewed studies on the association between lncRNA SNPs and overall cancer risk, and used the available data to perform a meta-analysis of 19 SNPs in five common lncRNA genes. The results suggest that the association between lncRNA SNPs and cancer risk can be categorized into four types: (i) complete association, where polymorphisms are significantly associated with risk of overall cancer in all genetic models, including ANRIL rs1333048, HOTTIP rs1859168, PRNCR1 rs16901946, PRNCR1 rs1016343, and PRNCR1 rs1456315; (ii) ANRIL rs4977574, ANRIL rs10757278, MALAT1 rs619586, HULC rs7763881, and PRNCR1 rs13252298 polymorphisms are only associated with cancer risk in some genetic models; (iii) no association, where the association of polymorphisms with cancer risk are not statistically significant, including ANRIL rs1333045 and PRNCR1 rs7007694; (iv) failed to be quantitatively synthesized due to limited studies. Therefore, the lncRNA SNPs provide more alternatives for biomarkers that can predict cancer risk.

More attention should be paid to several research directions in the future studies. First, more lncRNA polymorphisms and other aspects of cancer including chemotherapeutic susceptibility, metastasis, and relapse should be explored. Second, functional studies are needed to clarify the underlying mechanisms of lncRNA polymorphism in the tumorigenesis. Finally, the extensive clinical application of lncRNA polymorphisms requires further study.

## Supporting information

**Supplementary Table S1.** Quality assessment of eligible studies (Newcastle-Ottawa Scale).

**Supplementary Table S2.** The results of ORs and 95% CI of sensitivity analysis.

## Abbreviations

ANRIL: antisense non-coding RNA in the INK4 locus
CRS: created restriction site
HOTTIP: HOXA distal transcript antisense RNA
HULC: highly up-regulated in liver cancer
HWE: Hardy–Weinberg equilibrium
LD: linkage disequilibrium
lncRNA: long non-coding RNA
MALAT1: metastasis-associated lung adenocarcinoma transcript 1
ncRNA: non-coding RNA
NOS: Newcastle–Ottawa scale
OR: odds ratio
PRNCR1: prostate cancer-associated non-coding RNA 1
RFLP: restriction fragment length polymorphism
SNP: single-nucleotide polymorphism
T-ARMS: tetra-primer amplification refractory mutation system
95% CI: 95% confidence interval

## Appendix A: supplementary data

Supplementary data associated with this article can be found, in the online version.

Supplementary Table S1. Quality assessment of eligible studies NOS.

Supplementary Table S2. The results of ORs and 95% CI of sensitivity analysis.
