# Supplementary material for: Association between long non-coding RNA polymorphisms and cancer risk: a meta-analysis
Source: Biosci Rep. 2018 Jul 31;38(4):BSR20180365. doi: 10.1042/BSR20180365 (PMC6066654; doi:10.1042/BSR20180365)
Supplement: Supplementary file 1 [file bsr20180365_Supp1.pdf]

Supplementary Table S1. Quality assessment of eligible studies (Newcastle-Ottawa Scale).

Study	Selection				Comparability		Outcome		Total
	Adequacy of case definition	Number of case	Representativeness of the cases	Ascertainment of relevant cancers	Ascertainment of genotyping method	HWE	Assessment of outcome	Adequate follow up	
Khorshidi HR 2017	1	0	1	1	1	1	1	1	7
Kang M 2015	1	1	1	1	1	1	1	1	8
Taheri M 2017	1	0	1	1	1	1	1	1	7
Peng R 2017	1	1	1	1	1	1	1	1	8
Liu Y 2012	1	1	1	1	1	1	1	1	8
Li Y 2017	1	1	1	1	1	1	1	1	8
Gong WJ 2016	1	1	1	1	1	1	1	1	8
Hu P 2017	1	1	1	1	1	1	1	1	8
Shaker OG 2017	1	0	1	1	1	0	1	1	6
He BS 2017	1	1	1	1	1	1	1	1	8
Duan F 2017	1	1	1	1	1	1	1	1	8
Li L 2016	1	1	1	1	1	1	1	1	8
Sattarifard H 2017	1	1	1	1	1	0	1	1	7
Li L 2013	1	1	1	1	1	1	1	1	8
Chung S 2010	1	1	1	1	0	1	1	1	7
Salinas CA 2008	1	1	1	1	0	1	1	1	7
Zheng SL 2010	1	0	1	1	1	1	1	1	7

HWE, Hardy-Weinberg equilibrium.

Supplementary Table S2. The results of ORs and 95% CI of sensitivity analysis.

Excluding literature one by one	Allelic model<br>OR(95%CI)	Mutation homozygote vs. wild-type<br>OR(95%CI)	Heterozygote vs. wild-type<br>OR(95%CI)	Dominant model<br>OR(95%CI)	Recessive model<br>OR(95%CI)
ANRIL rs1333048 A/C					
Overall	**1.67(1.35-2.08)**	**2.40(1.60-3.59)**	**1.45(1.01-2.09)**	**1.79(1.28-2.51)**	**2.01(1.42-2.84)**
Hamid Reza Khorshidi 2017	**3.04(2.24-4.13)**	7.55(0.75-14.00)	2.98(0.73-5.13)	4.18(0.52-6.92)	3.93(0.38-6.50)
Mohammad Taheri 2017	0.88(0.64-1.21)	0.80(0.44-1.48)	0.69(0.40-1.18)	0.73(0.44-1.20)	1.01(0.61-1.69)
ANRIL rs4977574 A/G					
Overall	**0.76(0.64-0.97)**	0.72(0.43-1.18)	**0.62(0.44-0.87)**	**0.64(0.46-0.88)**	0.91(0.57-1.46)
Hamid Reza Khorshidi 2017	0.71(0.50-1.00)	0.58(0.28-1.21)	**0.61(0.37-0.99)**	**0.60(0.38-0.95)**	0.76(0.38-1.50)
Mohammad Taheri 2017	0.82(0.58-1.15)	0.87(0.43-1.74)	0.63(0.39-1.02)	0.68(0.42-1.07)	1.08(0.56-2.09)
ANRIL rs10757278 A/G					
Overall	**2.05(1.64-2.57)**	**4.26(2.67-6.78)**	**2.12(1.45-3.11)**	**2.57(1.80-3.69)**	**2.63(1.79-3.87)**
Hamid Reza Khorshidi 2017	**3.31(2.40-4.56)**	11.20(0.51-22.91)	4.74(0.49-9.03)	6.40(0.46-11.84)	3.94(0.36-6.57)
Mohammad Taheri 2017	1.25(0.90-1.71)	1.64(0.83-3.28)	1.21(0.73-2.00)	1.30(0.80-2.10)	1.47(0.79-2.73)
ANRIL rs1333045 C/T					
Overall	1.14(0.92- 1.43)	1.29(0.83-1.99)	1.03(0.71-1.49)	1.11(0.79-1.56)	1.29(0.89-1.88)
Hamid Reza Khorshidi 2017	1.00(0.73-1.37)	0.98(0.52-1.84)	1.09(0.67-1.80)	1.06(0.66-1.69)	0.93(0.53-1.63)
Mohammad Taheri 2017	1.31(0.95-1.81)	1.67(0.90-3.08)	0.96(0.55-1.66)	1.17(0.70-1.95)	**1.72(1.03-2.85)**
MALAT1 rs619586 A/G					
Overall	**0.77(0.65-0.91)**	0.58(0.28-1.19)	**0.78(0.65-0.94)**	1.19(0.87-1.62)	0.61(0.30-1.26)
Yao Liu 2012	**0.97(0.50-0.91)**	0.65(0.25-1.72)	**0.65(0.46-0.92)**	0.65(0.25-1.72)	0.70(0.26-1.85)
Rui Peng 2017	0.82(0.67-1.00)	0.51(0.17-1.50)	0.84(0.67-1.04)	0.51(0.17-1.50)	0.52(0.18-1.53)
HOTTIP rs1859168 A/C					
Overall	**1.31(1.19-1.45)**	**1.54(1.27-1.87)**	**1.24(1.06-1.45)**	**1.37(1.18-1.59)**	**1.49(1.26-1.76)**

Wei-Jing Gong 2016	**1.32(1.18-1.47)**	**1.54(1.24-1.90)**	1.18(0.99-1.41)	**1.34(1.14-1.57)**	**1.54(1.28-1.85)**
Pinghai Hu 2017	1.14(0.99-1.32)	1.15(0.87-1.51)	**0.76(0.60-0.97)**	0.93(0.75-1.16)	**1.53(1.21-1.93)**
Fujiao Duan 2017	**1.43(1.27-1.60)**	**1.83(1.51-2.45)**	1.72(0.44-2.06)	**1.77(1.50-2.10)**	**1.40(1.13-1.73)**
HULC rs7763881 A/C					
Overall	**0.90(0.82-0.99)**	0.86(0.71-1.04)	**0.74(0.63-0.86)**	**0.77(0.66-0.89)**	1.02(0.87-1.20)
Mingqiang Kang 2015	0.90(0.82-1.01)	0.87(0.70-1.08)	**0.75(0.63-0.89)**	**0.78(0.66-0.92)**	1.02(0.84-1.22)
Yao Liu 2012	0.86(0.72-1.03)	0.84(0.56-1.26)	**0.63(0.46-0.85)**	**0.66(0.50-0.89)**	1.05(0.74-1.49)
PRNCR1 rs16901946 G/A					
Overall	**1.15(1.06-1.24)**	**1.26(1.06-1.50)**	**1.15(1.02-1.28)**	**1.17(1.06-1.30)**	**1.21(1.03-1.43)**
Bang-Shun He 2017	**1.12(1.02-1.22)**	**1.21(1.01-1.45)**	1.11(0.98-1.25)	**1.13(1.01-1.27)**	1.17(0.99-1.39)
Lijuan Li 2016	**1.18(1.08-1.28)**	**1.36(1.14-1.63)**	**1.13(1.00-1.28)**	**1.18(1.06-1.33)**	**1.31(1.11-1.54)**
Suyoun Chung 2010	1.08(0.96-1.21)	1.01(0.80-1.29)	1.18(0.99-1.39)	1.15(0.98-1.34)	1.00(0.80-1.25)
PRNCR1 rs13252298 G/A					
Overall	**0.78(0.72-0.84)**	**0.67(0.56-0.81)**	**0.69(0.62-0.77)**	**0.81(0.73-0.90)**	0.85(0.72-1.01)
Bang-Shun He 2017	**0.76(0.71-0.84)**	**0.66(0.54-0.80)**	**0.67(0.59-0.76)**	**0.81(0.72-0.91)**	0.86(0.71-1.03)
Lijuan Li 2016	**0.74(0.68-0.80)**	**0.62(0.51-0.75)**	**0.63(0.56-0.71)**	**0.75(0.67-0.84)**	**0.82(0.69-0.98)**
Lijuan Li 2013	**0.78(0.72-0.85)**	**0.67(0.56-0.82)**	**0.69(0.61-0.78)**	**0.83(0.74-0.93)**	0.86(0.72-1.03)
Suyoun Chung 2010	0.95(0.85-1.07)	0.96(0.74-1.26)	0.87(0.74-1.01)	0.88(0.75-1.02)	1.14(0.89-1.46)
PRNCR1 rs7007694 C/T					
Overall	1.02(0.94-1.11)	1.19(0.98-1.44)	0.96(0.86-1.07)	0.99(0.89-1.10)	1.19(0.99-1.44)
Bang-Shun He 2017	1.01(0.93-1.11)	1.17(0.95-1.44)	0.94(0.83-1.06)	0.97(0.87-1.09)	1.17(0.96-1.44)
Lijuan Li 2016	1.07(0.98-1.16)	**1.27(1.03-1.54)**	0.99(0.89-1.12)	1.03(0.93-1.16)	**1.25(1.03-1.52)**
Hedieh Sattarifard 2017	1.04(0.96-1.13)	1.18(0.98-1.45)	0.98(0.87-1.09)	1.01(0.91-1.12)	1.19(0.99-1.44)
Lijuan Li 2013	1.01(0.93-1.10)	1.14(0.92-1.40)	0.95(0.84-1.07)	0.97(0.87-1.09)	1.14(0.94-1.39)
Suyoun Chung 2010	0.97(0.85-1.10)	1.16(0.80-1.67)	0.90(0.76-1.05)	0.92(0.79-1.08)	1.19(0.83-1.71)
PRNCR1 rs1016343 C/T					

Overall	**1.31(1.22-1.41)**	**1.66(1.41-1.97)**	**1.35(1.22-1.49)**	**1.41(1.28-1.55)**	**1.42(1.21-1.66)**
Lijuan Li 2016	**1.36(1.27-1.47)**	**1.86(1.55-2.22)**	**1.37(1.28-1.52)**	**1.45(1.31-1.59)**	**1.58(1.33-1.88)**
Lijuan Li 2013	**1.38(1.28-1.49)**	**1.88(1.57-2.26)**	**1.38(1.25-1.54)**	**1.46(1.32-1.61)**	**1.59(1.33-1.89)**
Claudia A. Salinas 2008	**1.30(1.19-1.41)**	**1.60(1.33-1.94)**	**1.36(1.21-1.54)**	**1.42(1.26-1.59)**	**1.35(1.13-1.61)**
Siqun Lilly Zheng 2010	**1.29(1.20-1.39)**	**1.61(1.35-1.91)**	**1.32(1.19-1.46)**	**1.37(1.25-1.51)**	**1.40(1.19-1.65)**
Suyoun Chung 2010	**1.20(1.09-1.32)**	**1.31(1.05-1.63)**	**1.30(1.14-1.48)**	**1.31(1.16-1.48)**	1.13(0.92-1.39)
PRNCR1 rs1456315 A/G					
Overall	**0.77(0.72-0.83)**	**0.59(0.50-0.69)**	**0.76(0.68-0.83)**	**0.72(0.66-0.79)**	**0.70(0.60-0.81)**
Lijuan Li 2016	**0.77(0.72-0.83)**	**0.60(0.51-0.71)**	**0.74(0.67-0.82)**	**0.71(0.65-0.78)**	**0.72(0.62-0.84)**
Lijuan Li 2013	**0.75(0.70-0.81)**	**0.54(0.46-0.64)**	**0.75(0.68-0.83)**	**0.71(0.64-0.78)**	**0.66(0.56-0.76)**
Suyoun Chung 2010	0.93(0.85-1.02)	**0.74(0.60-0.91)**	0.99(0.87-1.13)	0.96(0.84-1.08)	**0.80(0.67-0.97)**
Claudia A. Salinas 2008	**0.72(0.66-0.79)**	**0.48(0.39-0.60)**	**0.71(0.63-0.80)**	**0.68(0.61-0.76)**	**0.60(0.49-0.75)**

The results are in bold if the ORs and 95%CIs ranged from insignificantly to statistically significantly after removal of an individual study.
